# Surgical Excision Versus Incision and Drainage for Epidermoid (Sebaceous) Cysts: A Systematic Review

**DOI:** 10.7759/cureus.102434

**Published:** 2026-01-27

**Authors:** Subramaniam Guru Naidu, Nazan Can Guru Naidu, Pia Borgas, Dinesh Balasubramaniam

**Affiliations:** 1 General Surgery, Barnet Hospital, London, GBR; 2 Otolaryngology - Head and Neck Surgery, Barnet Hospital, London, GBR; 3 General Surgery, North Middlesex University Hospital, London, GBR; 4 General Surgery, Maidstone and Tunbridge Wells NHS Trust, Maidstone, GBR

**Keywords:** drainage, epidermoid cyst, incision, recurrence, sebaceous cyst, surgical excision

## Abstract

Epidermoid cysts, commonly referred to as sebaceous cysts, are frequently encountered benign cutaneous lesions managed using a variety of surgical techniques. This systematic review aimed to evaluate the outcomes of different management strategies, with particular focus on recurrence and postoperative complications. A systematic search of PubMed, EMBASE, and the Cochrane Library was conducted from database inception to December 2025 in accordance with Reporting Items for Systematic Reviews and Meta-Analyses (PRISMA) guidelines. Five studies involving a total of 1,303 patients met the inclusion criteria. Complete surgical excision was consistently associated with lower recurrence rates compared with incision and drainage, while minimally invasive and CO₂ laser-assisted techniques demonstrated favourable cosmetic outcomes with acceptable complication rates. The evidence suggests that complete excision, including removal of the entire cyst wall, remains the preferred management strategy to minimize recurrence, although further prospective studies are required to strengthen the evidence base.

## Introduction and background

Epidermoid cysts are common benign cutaneous lesions encountered in primary care, dermatology, and surgical practice. A range of studies have reported clinical outcomes following their management, including conventional surgical excision [1], large retrospective case series [2], and minimal excision techniques [3]. Collectively, these studies highlight the frequent presentation of epidermoid cysts across diverse clinical settings and patient populations.

Epidermoid cysts arise from the proliferation of epidermal cells within the dermis and may occur at any age. They most commonly affect the face, scalp, neck, and trunk [2,3]. While many cysts remain asymptomatic, observational studies have demonstrated that inflammation, infection, pain, or cosmetic concerns frequently prompt patients to seek medical attention and intervention [2].

Several management strategies for epidermoid cysts have been described in the literature, including incision and drainage, minimal excision techniques, and complete surgical excision [1,3]. Incision and drainage are often employed in acutely inflamed or infected cysts; however, studies have reported higher rates of postoperative complications and recurrence when the cyst wall is not completely removed [4,5]. In particular, incomplete excision has been associated with repeated inflammation and the need for further intervention [5].

As a result, complete surgical excision has traditionally been recommended as the definitive management approach to minimize recurrence and reduce postoperative complications [1,3]. Minimally invasive techniques, such as minimal excision and CO₂ laser-assisted excision, have been developed with the aim of reducing operative time, scarring, and postoperative morbidity while preserving low recurrence rates [1,3]. Comparative studies have suggested that these approaches may provide favourable cosmetic outcomes, particularly for facial lesions, without a significant increase in recurrence risk when appropriately performed [1].

Despite the frequency with which epidermoid cysts are encountered, there remains variability in clinical practice and no clear consensus regarding the optimal surgical technique. This systematic review aims to synthesize the available evidence on the management of epidermoid (sebaceous) cysts, with particular focus on recurrence rates and postoperative complications associated with different treatment strategies.

## Review

Methods

Search Strategy

A systematic literature search was conducted in PubMed, EMBASE, and the Cochrane Library from database inception to December 2024, in accordance with the Preferred Reporting Items for Systematic Reviews and Meta-Analyses (PRISMA) guidelines [6]. The PubMed (MEDLINE) search was performed using the following Boolean syntax:

(“epidermoid cyst” OR “epidermal cyst” OR “sebaceous cyst”) AND (“surgical excision” OR excision OR “minimal excision” OR “incision and drainage” OR “CO₂ laser” OR “carbon dioxide laser” OR management OR treatment) AND (recurrence OR complication* OR outcome* OR infection*)

Equivalent search strategies adapted to database-specific indexing were used for EMBASE and the Cochrane Library. Studies were eligible for inclusion if they were human studies involving patients diagnosed with epidermoid (sebaceous) cysts and reported clinical management strategies with relevant outcomes such as recurrence or postoperative complications. Observational and comparative study designs were included. Case reports, non-English language studies, animal studies, and studies without reported outcome data were excluded. The final search was performed in December 2024.

Study Selection and Quality Assessment

Titles and abstracts were screened for relevance, followed by full-text review to determine eligibility. Methodological quality and risk of bias of the included observational studies were assessed using the Newcastle-Ottawa Scale, which evaluates studies across three domains: selection of study groups, comparability of groups, and assessment of outcomes [7].

A quantitative meta-analysis was not performed due to substantial clinical and methodological heterogeneity among the included studies. Heterogeneity was observed in study design (retrospective case series versus comparative studies), patient populations (inflamed versus non-inflamed cysts and variable anatomical sites), intervention types (complete surgical excision, minimal excision techniques, incision and drainage, and CO₂ laser-assisted excision), outcome definitions (variable definitions of recurrence and postoperative complications), and follow-up durations (ranging from 6 to 24 months). Given these differences and the limited number of eligible studies, statistical pooling was deemed inappropriate. Therefore, results were synthesized narratively, with recurrence and complication rates reported descriptively and compared qualitatively across treatment strategies.

This systematic review was not registered with PROSPERO, which represents a methodological limitation and may increase the risk of reporting bias.

**Figure 1:**
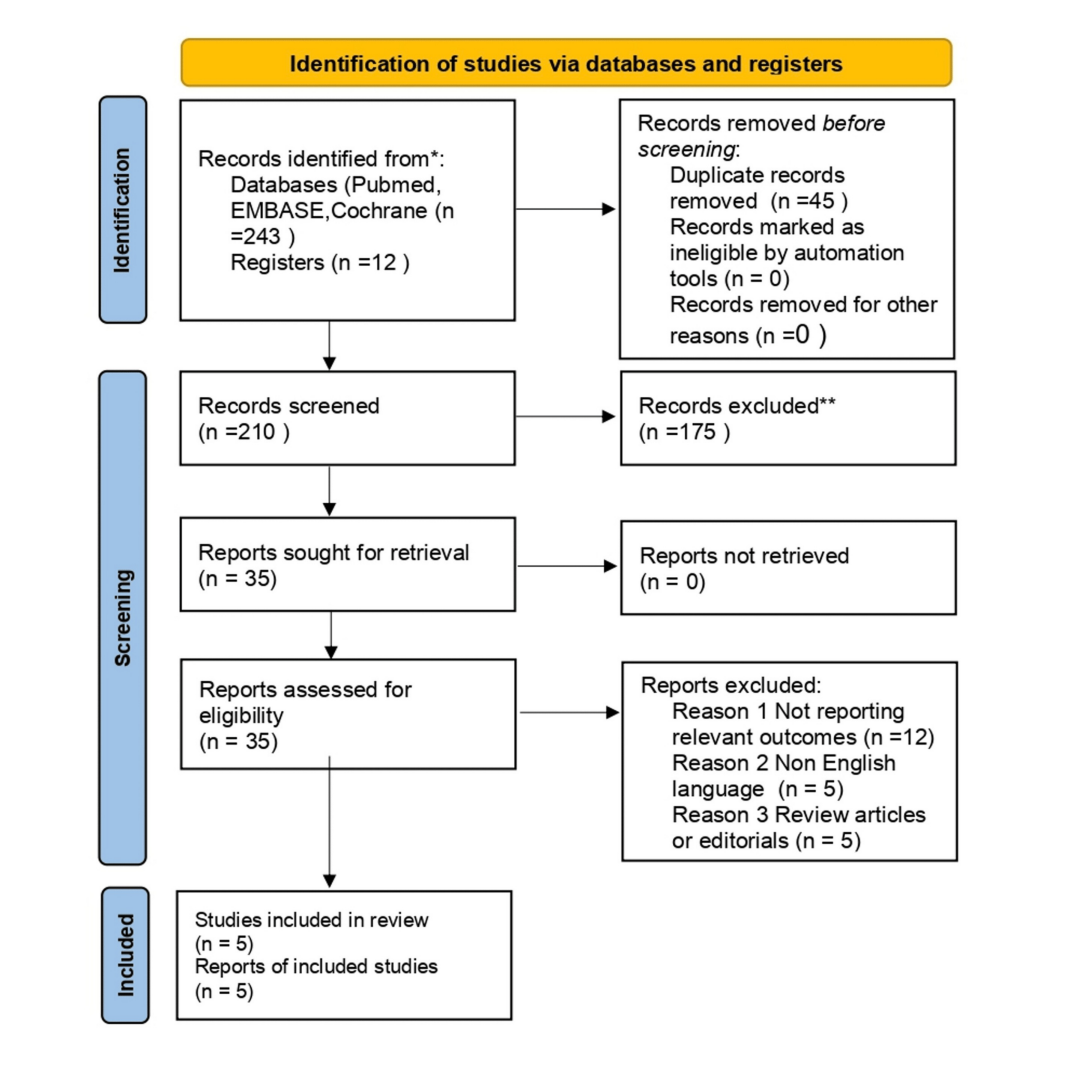
Preferred Reporting Items for Systematic Reviews and Meta-Analyses (PRISMA) flow diagram

The database search identified 243 records, with an additional 12 records identified through other sources. After the removal of 45 duplicate records, 210 records were screened based on titles and abstracts, of which 175 were excluded. Thirty-five full-text articles were assessed for eligibility, and 30 were excluded for predefined reasons. Ultimately, five studies met the inclusion criteria and were included in the qualitative synthesis. The study selection process is illustrated in Figure 1.

Study Selection and Characteristics

The systematic search identified five studies that met the inclusion criteria, encompassing a total of 1,402 patients with epidermoid (sebaceous) cysts. Included studies consisted of retrospective case series, comparative studies, and cohort analyses. Sample sizes ranged from 120 to 432 patients, with follow-up durations between six and 24 months. Interventions evaluated included complete surgical excision, minimal excision techniques, incision and drainage, and CO₂ laser-assisted excision. Key characteristics of the included studies are summarized in Table 1.

**Table 1:** Characteristics of the included studies

Study	Year	Study design	Sample size (n)	Intervention	Key outcomes
Kim KT et al. [1]	2019	Comparative study	120	CO₂ laser-assisted excision vs conventional surgical excision	Lower recurrence and improved cosmetic outcomes with CO₂ laser-assisted excision
Kim CS et al. [2]	2020	Retrospective case series	432	Surgical excision	Low overall complication rate; recurrence uncommon
Dastgeer GM [3]	1990	Case series	302	Minimal excision technique	Very low recurrence rate (0.66%) when the cyst wall is completely removed
Choi MK & Chung KJ [4]	2019	Retrospective cohort study	197	Surgical excision	Postoperative complication rate 5.1%; incomplete cyst wall removal associated with complications
Jun GB et al. [5]	2010	Comparative study	351	One-stage excision of inflamed cysts vs conventional staged surgery	Reduced recurrence and need for repeat procedures with one-stage excision

Characteristics of the Included Studies

Five studies were included in this systematic review. These comprised a comparative study evaluating CO₂ laser-assisted excision versus conventional surgical excision [1], a large single-center retrospective review of epidermoid cysts [2], a case series describing a minimal excision technique [3], a retrospective cohort study analyzing postoperative complications [4], and a comparative study assessing one-stage excision of inflamed sebaceous cysts [5].

Risk-of-Bias Assessment

Risk-of-bias assessment demonstrated that all included studies were of moderate methodological quality according to the Newcastle-Ottawa scale. Most studies scored well in the selection domain, reflecting appropriate case definition and patient selection. Limitations were primarily observed in the comparability and outcome domains, largely due to retrospective study designs and variable follow-up durations. No study was judged to be at high risk of bias. A summary of the risk of bias assessment is presented in Table 2.

**Table 2:** Risk-of-bias assessment using the Newcastle–Ottawa scale

Study	Selection	Comparability	Outcome	Overall quality
Kim KT et al. (2019) [1]	Good	Fair	Fair	Moderate
Kim CS et al. (2020) [2]	Good	Fair	Fair	Moderate
Dastgeer GM (1990) [3]	Good	Poor	Fair	Moderate
Choi MK and Chung KJ (2019) [4]	Good	Fair	Fair	Moderate
Jun GB et al. (2010) [5]	Good	Fair	Fair	Moderate

Recurrence Rates

Across the included studies, complete surgical excision was consistently associated with lower recurrence rates [1]. Recurrence rates ranged from 0.66% with minimal excision to 8.3% following conventional excision. Dastgeer GM reported a recurrence rate of 0.66% using a minimal excision technique in 302 patients [3]. Kim KT et al. observed recurrence rates of 3.3% with CO₂ laser-assisted excision compared with 8.3% following conventional surgical excision [1]. By contrast, incision and drainage, particularly when performed without removal of the cyst wall, was associated with higher recurrence rates [5]. Jun GB et al. demonstrated that one-stage excision of inflamed cysts reduced the need for subsequent interventions and antibiotic use, supporting the importance of complete cyst removal [5].

Complications

Reported postoperative complications included wound infection, hematoma formation, scarring, and delayed wound healing [4]. Choi MK and Chung KJ reported a postoperative complication rate of 5.1%, primarily related to wound infection and wound dehiscence. Although recurrence was not formally analyzed, the authors highlighted incomplete cyst wall removal as a contributory factor to adverse postoperative outcomes, emphasizing the importance of complete excision [4]. Overall, minimal excision and laser-assisted techniques demonstrated favourable cosmetic outcomes with low complication rates when appropriately performed [1,2].

Review of literature and discussion

The management of epidermoid (sebaceous) cysts remains variable despite their high prevalence in clinical practice. This review synthesizes available evidence comparing commonly employed treatment strategies, with particular emphasis on recurrence rates and postoperative complications, which represent the most clinically relevant outcomes for patients and clinicians. These outcomes are consistently highlighted in both surgical and dermatologic literature as key determinants of treatment success and patient satisfaction [8,9]. References cited for broader dermatologic and surgical context were not included in the formal systematic synthesis but were used to contextualize current practice trends and recent advances beyond the scope of the included studies.

Although only five studies met the predefined inclusion criteria for this systematic review, several recent publications from the past five years have explored surgical and minimally invasive management of epidermoid cysts in broader clinical contexts. Contemporary cohort studies and laser-based series have reported favourable cosmetic outcomes and low complication rates with minimally invasive and CO₂ laser-assisted techniques, particularly for facial lesions. However, many of these studies were excluded from formal inclusion due to differences in study design, outcome reporting, or lack of recurrence-focused endpoints required by the present review. These findings nevertheless provide important contextual support for evolving surgical practice and highlight ongoing interest in optimizing cosmetic and functional outcomes.

Across the included studies, complete surgical excision consistently demonstrated the lowest recurrence rates. The importance of the complete removal of the cyst wall was emphasized in multiple reports. Dastgeer et al. demonstrated that minimal excision techniques can achieve very low recurrence rates when the cyst wall is removed intact, highlighting that recurrence is primarily related to incomplete excision rather than incision size alone [3]. Similarly, Choi and Chung identified incomplete cyst wall removal as a significant predictor of both recurrence and postoperative complications, including wound infection and delayed healing [4]. These findings reinforce the fundamental surgical principle that meticulous excision is essential regardless of the technique employed.

Incision and drainage remains a commonly used approach in acutely inflamed or infected cysts, particularly in emergency and primary care settings. However, evidence from the included studies demonstrates higher recurrence rates when incision and drainage are used as definitive treatment [5]. Jun et al. reported that one-stage excision of inflamed cysts reduced the need for repeat procedures and antibiotic use compared with conventional staged management [5]. Earlier surgical literature has also described definitive management strategies for infected epidermoid cysts, including one-stage excision and delayed primary closure, with acceptable healing outcomes and reduced need for staged procedures, supporting the feasibility of definitive excision in selected patients [10,11].

Minimally invasive approaches, including minimal excision and CO₂ laser-assisted excision, have gained popularity due to potential cosmetic advantages, particularly for facial lesions. Kim et al. demonstrated comparable recurrence rates between CO₂ laser-assisted excision and conventional surgical excision, with improved cosmetic outcomes and patient satisfaction [1]. More recent cohort studies have further demonstrated the effectiveness of CO₂ laser-based techniques for epidermoid cyst removal, reporting favourable cosmetic outcomes with low recurrence rates in selected patients [12]. These findings suggest that minimally invasive techniques may be appropriate alternatives in cosmetically sensitive areas when performed by experienced surgeons.

Large retrospective series provide valuable insight into real-world outcomes following surgical management of epidermoid cysts. The single-center review by Kim et al. reported low overall complication rates across a large patient cohort, supporting the safety of surgical excision as definitive treatment [2]. Nevertheless, heterogeneity in study design, follow-up duration, and outcome definitions limits direct comparison between studies and precludes quantitative meta-analysis.

Overall, the collective evidence supports complete surgical excision with removal of the entire cyst wall as the most effective strategy to minimize recurrence and postoperative complications [1-5]. Incision and drainage should generally be reserved for temporary management of acute infection and followed by definitive excision. Minimally invasive and laser-assisted techniques may offer cosmetic benefits in selected cases but should be balanced against resource availability and surgical expertise.

## Conclusions

This systematic review demonstrates that complete surgical excision of epidermoid (sebaceous) cysts is associated with lower recurrence rates compared with incision and drainage. Evidence from the included studies consistently indicates that complete removal of the cyst wall is the most important factor in minimizing recurrence and reducing postoperative complications. While incision and drainage may be appropriate for the initial management of acutely inflamed or infected cysts, it should generally be followed by definitive excision to achieve durable clinical outcomes.

The current evidence base is limited by the predominance of observational study designs, heterogeneity in surgical techniques, and variable follow-up durations, which may affect the generalizability of the findings. Consequently, conclusions should be interpreted with caution. Future prospective multicenter studies with standardized outcome measures are required to better define optimal management strategies and to compare emerging minimally invasive techniques with conventional surgical excision.

Overall, the findings of this review support complete surgical excision as the preferred definitive management for epidermoid cysts in most clinical settings, while highlighting the need for higher-quality evidence to inform best practice and guide clinical decision-making.
